# Vibration induced refrigeration using ferroelectric materials

**DOI:** 10.1038/s41598-019-40159-8

**Published:** 2019-03-08

**Authors:** Anuruddh Kumar, Aditya Chauhan, Satyanarayan Patel, Nikola Novak, Rajeev Kumar, Rahul Vaish

**Affiliations:** 1 School of Engineering, Indian Institute of Technology Mandi, Mandi, H.P 175 001 India; 2 Department of Materials Science and Metallurgy, University of Cambridge, Cambridge, CB3 0FS UK; 3 Institute of Materials Science, Technische Universität Darmstadt, 64287 Darmstadt, Germany

## Abstract

This article aims to propose a cantilever based cooling device employing non-axis symmetric placement of bulk ferroelectric patches. Ambient mechanical vibrations produce large stresses in cantilevers resulting in elastocaloric effect associated with ferroelectrics. Further, design allows cascading of several cantilevers to achieve large cooling response. A finite element analysis of the system was performed using material properties of bulk 0.50Ba(Zr_0.2_Ti_0.8_)O_3_−0.50(Ba_0.7_Ca_0.3_)TiO_3_. An individual element could produce a peak elastocaloric effect of 0.02 K (324 K); whereas the proposed system could achieve a temperature drop of 0.2 K within 50 seconds (10 elements, 1.5 Hz). Furthermore, net cooling can be further improved about ~2 K (using 10 cantilevers) for a starting temperature of 358 K. This study shows that elastocaloric effect in ferroelectric materials is capable of converting waste mechanical vibration into refrigeration effect which is not reported so far.

## Introduction

Development of suitable solid-state cooling technology can potentially usher in a new generation of efficient, eco-friendly and miniaturized refrigeration architecture. This is especially important for on-board thermal management of mobile electronic devices, where temperature regulation is a priority. In this regard, several techniques are being developed simultaneously including Peltier cooling and ferroic caloric effects (electrocaloric, elastocaloric and magnetocaloric)^1–5^. The last decade has witnessed commercialization of Peltier cooling and advent of magnetocaloric prototypes^6^. It is plausible to assume that other solid-state technologies will soon transcend from laboratory trials to prototypes and field tests. Peltier cooling already holds the status of established technologies with competitive products available^7,8^. However, drawbacks like low efficiency and poor temperature regulation are still present. Hence, ferroic caloric effects are being developed to obtain a fast and responsive cooling higher efficiency. Ferroelectric based refrigeration seems to be the frontrunner in this regard[1]. The sheer volume of research being published in this direction indicates the vested interest towards commercial development of this technology^2,3,9–11^. However, major effort in this field has been concentrated towards increasing the upper limit of caloric capacity^2,3,10,12^. Alternately, little effort has been made towards the design of a practical refrigerator/heat exchanger for onboard installation^13–15^. Bruederlin *et al*.^13^ discussed the major challenges in design and fabrication of SMA film-based elastocaloric cooling devices and proposed an elastocaloric cooling devices. Ma *et al*.^14^ proposed an electrocaloric cooling devices which is attached with electrostatic actuation mechanism to produce cooling effect by allowing efficient heat transfer through good thermal contacts with the heat source or heat sink.

An ideal refrigeration system should also be able to employ renewable energy. In this regard, a large number of systems have been tested and reported with promising results. These are all based on vapor absorption, solar-thermal and thermoelectric principles^16–21^. Cooling through waste energy could be another potential topic of research which has not been explored so far. Particularly, utilization of ambient mechanical vibrations to produce cooling effect can be an interesting topic. In this article, ferroelectric based refrigeration has been proposed which utilizes ambient vibrations to produce the required cooling effect through elastocaloric effect. Through careful selection of operating parameters, this cantilever-based device has been designed to induce significant stress in piezoelectric material. This in turn allows manifestation of large elastocaloric effects in bulk material, which is used as a heat exchanger system. The overall operation and performance of the same has been evaluated using finite element analysis. The ferroelectric material selected for the study is bulk lead-free 0.50Ba(Zr_0.2_Ti_0.8_)O_3_−0.50(Ba_0.7_Ca_0.3_)TiO_3_ (50BZT-50BCT). An individual element comprises of a cantilever of dimensions (25 × 5 × 0.1 mm^3^) where 50BZT-50BCT patch is installed near fixed end of cantilever. Further details and analysis have been described in the following sections.

## Materials and Methods

0.50Ba(Zr_0.2_Ti_0.8_)O_3_−0.50(Ba_0.7_Ca_0.3_)TiO_3_ (50BZT-50BCT) represents a solid solution of two individual ferroelectric phases namely Barium Zirconate Titanate and Barium Calcium Titanate. BZT-BCT based compositions have been recently explored as promising lead-free ferroelectrics with enhanced piezo/pyroelectric coefficients and a low saturation electric field. Further, this ratio (50BZT-50BCT) lies in the vicinity of a polymorphic phase boundary (PPB). Presence of a PPB further improves ferroelectric performance and has been reported for large reversible strain, corresponding to phase transition^22^. Hence, it forms suitable material for this study.

Due to lack of pre-existing data, a polycrystalline ceramic sample was prepared and its properties were measured. The material was prepared by the conventional solid-oxide reaction route. Raw powder powders of BaCO_3_ (99.8%), CaCO_3_ (99.5%), ZrO_2_ (99.5%), and TiO_2_ (99.6%) (all from Alfa-Aesar GmbH & Co., Karlsruhe, Germany) were mixed in stoichiometric ratios and ball milled for 5 hours. The milled powders were calcined in alumina crucible for 2 hours at 1300 °C in air. The calcined powders were milled again and pressed into cylinders sample of size 10 mm × 6 mm (diameter × height) using a cold isostatic press (CIP 100 E, Paul-Otto Weber GmbH, Remshalden, Germany) at 300 MPa and sintered at 1500 °C in air for 2 hours. Powder X-ray diffraction analysis was used to confirm crystallization and phase formation of sintered pellets. Scanning electron microscopy (SEM) was used to obtained microstructural and surface information and density was confirmed using Archimedes principle. These characterization methods indicated the phase purity of sintered pellets and confirmed high density (5.6 g/cm^3^); with a uniform microstructure and free of any major porosity. The flat faces were polished and stress-strain behavior was obtained using a screw-driven load frame (Z010, Zwick GmbH & Co. KG, Ulm, Germany). A compressive pre-stress of 5 MPa was applied on the sample to assure a smooth contact (without losing contact to the sample) using a force control mode. Through this, stress-strain loops were generated at different operating temperatures (304 K to 380 K). Once this data has been obtained, the elastocaloric effect of the material can be calculated.

### Elastocaloric effect in ferroelectric materials

The elastocaloric ∆T for any material can be determined using the following expression^23^:

1 $${\rm{\Delta }}T=-\frac{T}{\rho \ast {c}_{p}}{\int }_{{\sigma }_{1}}^{{\sigma }_{2}}(\frac{\partial \in }{\partial T})d\sigma $$

Here the symbols represent their usual quantities of density (*ρ*), specific heat capacity (*c*_*p*_), strain ($\in$), stress (*σ*) and temperature (T). Equation (1) represents a modified form of the classical Maxwell’s relation for calculating entropy change within a ferroic material. This is an indirect method used to evaluate the expected temperature change and is subject to certain limitations discussed elsewhere^24,25^. Nevertheless, this approach is helpful in providing a qualitative estimate of the caloric capacity for the material in question. Upon substituting the appropriate material parameters in Eq. (1), the elastocaloric effect can be estimated for 50BZT-50BCT.

### Modeling of the refrigeration process

In present study, mechanical vibration and heat transfer are the main domains for calculating the net cooling effect. To simulate this, finite element analysis has been used as this approach is widely accepted due to its good agreement with experimental studies. Shell element has been employed for modelling the cantilever, as the third dimension of the cantilever makes the structure thin. In finite element formulation of shell type structure, linear elastic theory has been employed. Each element consists of three translational degrees of freedom (*u*_*oi*_, *v*_*oi*_, *w*_*oi*_) and two rotational degree of freedom (*α*_*i*_*β*_*i*_ and *β*_*i*_). Displacement at any point ‘P’ inside the k^th^ layer within the element can be written as:

2 $$\{\begin{array}{c}u\\ v\\ w\end{array}\}=\sum _{i=1}^{nnel}{N}_{i}\{\begin{array}{c}{u}_{0i}\\ {v}_{0i}\\ {w}_{0i}\end{array}\}+\sum _{i=1}^{nnel}{N}_{i}{H}_{i}\{\begin{array}{cc}{l}_{1i} & -{l}_{2i}\\ {m}_{1i} & -{m}_{2i}\\ {n}_{1i} & -{n}_{2i}\end{array}\}\times \{\begin{array}{c}{\alpha }_{i}\\ {\beta }_{i}\end{array}\}$$

Where *l*_1*i*_, *m*_1*i*_ and *n*_1*i*_ are the direction cosine of tangent unit vector *V*_1*i*_; and *l*_2*i*_, *m*_2*i*_ and *n*_2*i*_ are the direction cosine of tangent unit vector *V*_2*i*_ at node *i*. Therefore, each node has three translational degrees of freedom (*u*_*oi*_, *v*_*oi*_, *w*_*oi*_) in the global coordinates and two rotational degrees of freedom (*α*_*i*_ and *β*_*i*_) about local coordinate. Further, *N*_*i*_ is the shape function at node *i*.

The strain vector *ε* is defined by the first partial derivative of the displacement vector [*uvwαβ*]^*T*^ by using a differential operator matrix as follows:

3 $$\{\varepsilon \}=\{\begin{array}{c}{\varepsilon }_{x}\\ {\varepsilon }_{y}\\ {\varepsilon }_{z}\\ {\gamma }_{xy}\\ {\gamma }_{yz}\\ {\gamma }_{zx}\end{array}\}=\{\begin{array}{c}\frac{\partial u}{\partial x}\\ \frac{\partial v}{\partial y}\\ \frac{\partial w}{\partial z}\\ \frac{\partial u}{\partial y}+\frac{\partial v}{\partial x}\\ \frac{\partial v}{\partial z}+\frac{\partial w}{\partial y}\\ \frac{\partial w}{\partial x}+\frac{\partial u}{\partial z}\end{array}\}={[B]}_{e}{\{q\}}_{e}\,{\rm{where}}\,{\{q\}}_{e}=\{\begin{array}{c}{u}_{oi}\\ {v}_{oi}\\ {w}_{oi}\\ {\beta }_{i}\\ {\alpha }_{i}\end{array}\}$$

Further, using the Hamilton’s principle, the equation of motion for the shell element can be written as^26^:

4 $${\int }_{{t}_{1}}^{{t}_{2}}(\delta K{E}^{e}-\delta {U}^{e}+\delta {W}_{ext})dt=0$$

Where, *KE* is kinetic energy, *U* is potential energy and *W* is work done by external forces. Using Hamilton’s principle, the governing equation for an element can be written as;

5 $${[M]}_{e}{\{\ddot{q}\}}_{e}{[C]}_{e}\{\dot{q}\}+{[K]}_{e}{\{q\}}_{e}=\{F\}$$

Where [*M*], [*C*] and [*K*] are mass matrix, damping matrix and stiffness matrix of the cantilever and {*F*} is applied mechanical force or vibration. This equation was solved using 4^th^ order Runge Kutta method to estimate the cantilever response in time series, followed by post processing to compute the stresses in the cantilever. For the simulations 50BZT-50BCT ceramics and platinum host layer materials properties are used as follow: Young’s modulus is 88 and 168 GPa, poisson ratio is 0.3 and 0.28, density 5600 and 21450 kg/m^3^, respectively.

Details of finite element formulation are mentioned in the Supplementary File.

Next, the heat transfer between the cantilevers during contact was investigated. Under tensile stress the material heats up while cooling is observed under compressive stress. The behavior can be described as:

6 $${Q}_{ELC}=\{\begin{array}{c}m{c}_{p}{\rm{\Delta }}T\,\,\,\,\,if\sigma  > 0\\ -m{c}_{p}{\rm{\Delta }}T\,\,\,\,\,if\sigma  < 0\end{array}$$

Where, *m* is mass of cantilever, *c*_*p*_ is specific heat capacity, and Δ*T* is temperature change during vibrations. In the operation process, alternate cantilevers move in opposite phases such that upon contact unidirectional heat transfer can be achieved. We have not considered any type of conduction loss or heat loss in the system. Validation of both formulations dynamic and heat transfer studies has been given in the Supplementary File which shows the good agreement with literature results.

## Results and Discussion

**Figure 1:**
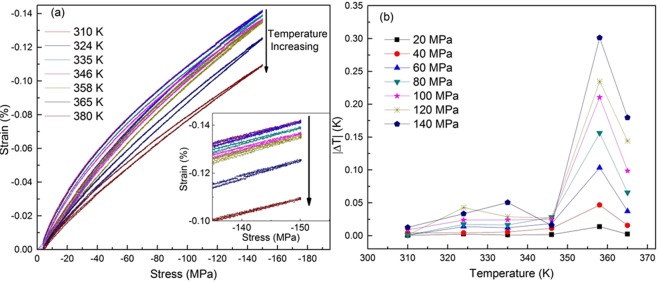
(**a**) stress-strain plots of 50BZT-50BCT as a function of temperature and (**b**) corresponding elastocaloric effect. Inset shows enlarge view of stress-strain plot.

In order to determine the caloric effect, the slope of stress-strain plot with respect to temperature needs to be established. This data has been provided in Fig. 1(a), where the graph demonstrates the stress-strain behavior in compression mode, as a function of temperature. Specific heat capacity of the material as a function of temperature (un-stressed) which is adapted from^27^. Further, owing to the low compressibility of solids it is plausible to assume that any associated change would be very small and can be safely ignored. Data from Fig. 1 indicates that progressive increase in temperature leads to a concomitant increase in Young’s modulus. This is typical of a ferroelastic material and the resultant strain continually decreases upto a temperature of 358 K, then drop is observed at 365 K. This data (Fig. 1a) has been used in conjunction with Eq. (1) to generate the plots for elastocaloric effect as a function of applied stress and temperature, presented in Fig. 1(b). It can be visualized from the figure that upto an operating temperature of 346 K, the caloric effect remains negligible after which increases from 0.15 K to 0.3 K (358 K) for an applied stress of 140 MPa. This can be attributed to the slow increment in the value of specific heat, while strain remains almost constant. Hence, the temperature increases (0.15 K to 0.3 K) in caloric capacity at 358 K can be attributed to the comparatively larger strain. It can be proposed that beyond 358 K, a phase transition is initiated which results in large reversible strain in the material when subjected to compressive stress^22,27–29^. However, *in-situ* X-ray analysis is needed to confirm this hypothesis. In the Fig. 1(b), it is important to note that the maximum elastocaloric obtained near Curie temperature and on the other hand Curie temperature can be shifted with the stress. In this context, Schader *et al*. demonstrated influence of uniaxial stress on the ferroelectric-to-paraelectric phase change in BaTiO_3_^30^. It was demonstrated that the applied stresses can decrease Curie temperature by ~5 °C (at 150 MPa). Therefore, similar behavior can be expected in the present work. However, our measurement step is large ~10 °C. Hence, we did not observe this phenomenon. If we record data at every 1 °C step or less in the vicinity phase transition then it is possible to observe Curie temperature behavior. Due to experimental constraints and out of the scope from the present work, this phenomenon is not discussed further. Nevertheless, to utilize this elastocaloric effect, a simple arrangement of cantilevers is proposed which employs ambient vibrations to drive and deliver the cooling between hot (sink) and cold (source) bodies.

**Figure 2:**
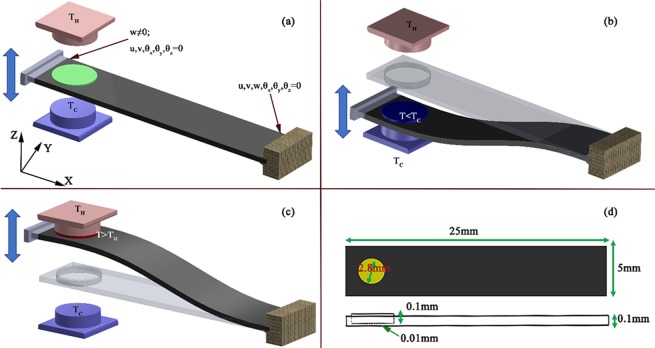
(**a**–**c**) Schematic of the proposed cooling technique with a single cantilever (**d**) dimensions of the cantilever.

Figure 2 describes the schematic of the proposed cooling technique with a single cantilever. The setup consists of two finite mass bodies (hot and cold) with a cantilever arrangement between them as shown in Fig. 2(a). This is one of the simplest designs to convert mechanical vibration into cooling effect. Cantilever consists of 50BZT-50BCT circular patch (diameter = 2.8 mm, thickness = 0.1 mm) at fixed end having platinum as host layer. Total thickness of the host layer with patch is 0.11 mm, while thickness of host layer at the patch is 0.01 mm. Hence, effective stress in 50BZT-50BCT patch either in tension or compressive states without affecting the heat transfer as the host layer thickness is negligible for heat transfer. The curvature of the source and sink allows for effective area-contact necessary for the heat exchange. In order to have a large cooling effect, material should experience large stresses as ΔT is directly proportion to the stress. Therefore, to generate maximum stresses, one end of cantilever is fixed while its other end is clamped and vibrated with fixed frequency (1.5 Hz) and amplitude (2 mm) as shown in Fig. 2(a). This amplitude deflects the cantilever resulting in an effective stress near the fixed end of the cantilever in Fig. 2(b,c). It is to be noted that as the cantilever deflects from one direction to the other, a symmetric arrangement would cause both tensile and compressive stresses to develop simultaneously which would neglect any caloric effect. Hence, to have a meaningful exchange of thermal energy, the piezoelectric materials are kept outside the neutral axis of the structure. This enables cycling of the material between compressive and tensile stresses thereby yielding systematic heating and cooling of the material. It is to be noted that within a complete cycle from tension (σ) to compression (-σ), effective ∆T is doubled as compared to that observed for one half of a cycle (0 to σ). Figure 2(b) shows the deformed shape of the cantilever when patch is in compression stresses and make a contact with cold body. In this stage, temperature of the patch goes down to below the cold body and makes a heat transfer from cold body to patch. Figure 2(c) shows deformed shape of cantilever in opposite phase in which patch has tension stresses due to this temperature is higher than hot body and transfer the heat from patch to hot body. Dimensions of the cantilever and patch are shown in Fig. 2(d).

**Figure 3:**
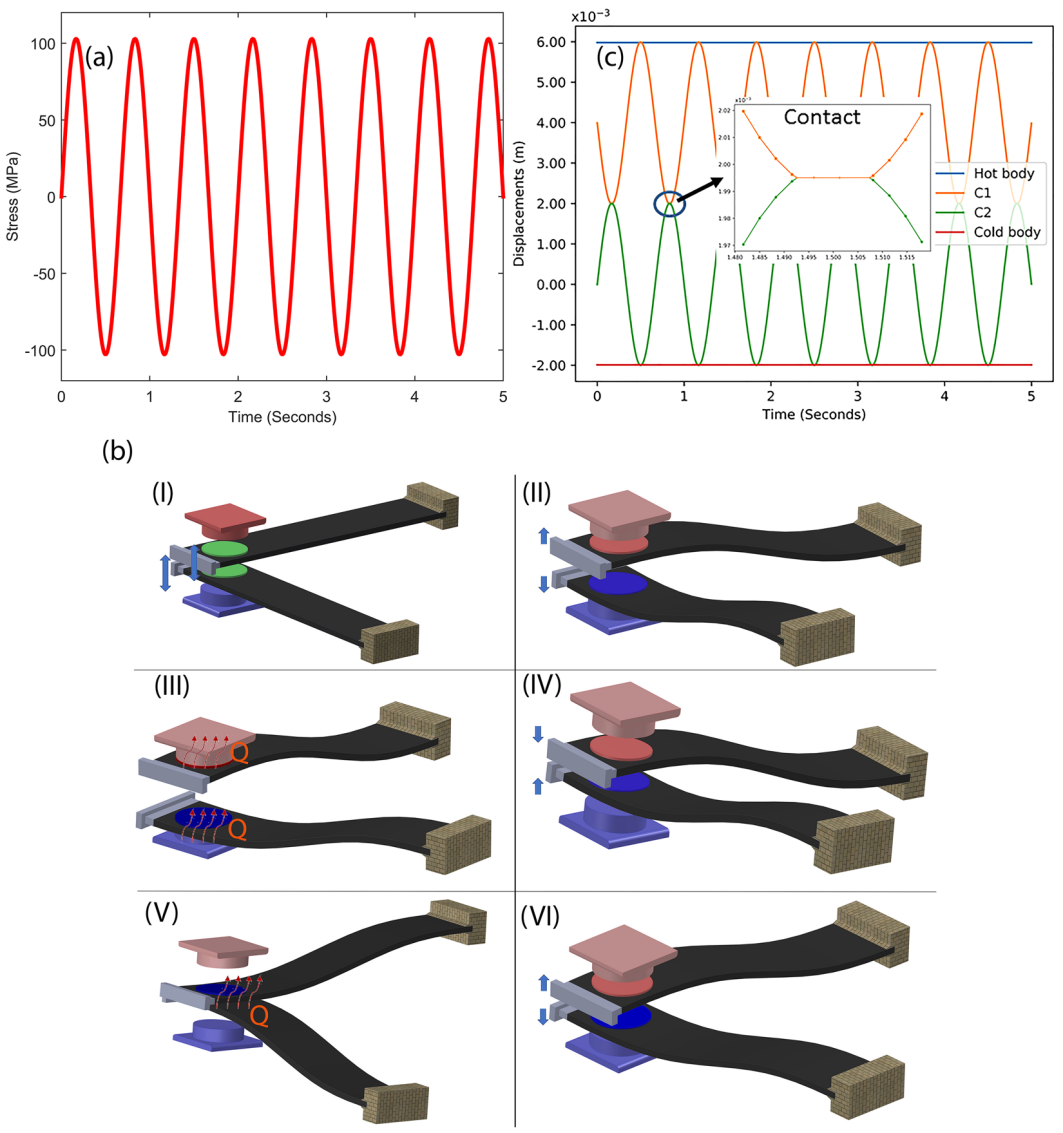
(**a**) Stress developed in the cantilever during vibration. (**b**) Displacement and contact between two cantilevers (C1 and C2) during vibration (**c**) cascaded schematic of working mechanism of heat transfer during cantilevers in motion.

Figure 3(a) displays the variation of stress in the beam as a function of time. The selected dimensions of the cantilever allow sustainable generation of high intensity stress (100 MPa) without affecting long-term performance. The overall operation mechanism is similar to an Ericsson cycle, where the material is first cooled below source temperature to extract energy and then heated above sink temperature to refrigeration process^31,32^. However, given the limited ∆T of 0.21 K at 100 MPa and 358 K, a single element would take too long to produce any considerable cooling effect apart from very low load conditions. To address this problem, we modified the approach to include two cantilevers which were oriented at an angle of 90° from each other as shown in Fig. 3(b). Figure 3(b) has been used to explain the cooling mechanism using two cantilevers. It is clear from the figure that two cantilevers are able to transfer heat from lower temperature body to higher temperature body when these cantilevers deflect in 180 degree phase difference. Initially, both the cantilevers make contact with either the source or sink and then exchange energy by making contact with each others. It is to note that there is slight difference in loading conditions (Figs 1 and 2). In Fig. 1, mechanical loading has been implemented in the direction on poling. However, in cantilever geometry (Fig. 2), mechanical loading is perpendicular to poling. Such conditions can deviate cooling performance upto some extent. In view of neither experimental nor theoretical work has been done to observe mechanical loading direction dependent elastocaloric effect in ferroelectric materials, we have used data from Fig. 1 to explain cantilever-based vibration driven refrigeration device.

This cascaded setup allows each cantilever to move independently without obstructing the vibration of other beams, while still providing effective area of contact for effective heat exchange. The setup itself is graphically depicted the stage (I to VI) in Fig. 3(b). Step I is the initial stage where both cantilevers are un-deformed and temperature of the patches are equal. Second step (II) represents the stage when cantilevers vibrate in 180 degree phase difference so that one ferroelectric patch starts to cool and another patch (stick on other cantilever structure) starts to hot. In third stage (III), one cantilever having cold patch makes the contact with cold body and absorbed heat from the cold body due to their temperature deference. Similarly, other cantilever makes the contact with the hot body and transfer the heat to the sink. In step IV, cantilevers move in opposite direction and make contact with each other as shown in stage (V) and transfer the heat from hot patch to the cool patch. After this, cantilevers separate and start to move in opposite direction as shown in stage (VI). This process can be repeated to provide the necessary cooling effect in cycle.

Figure 3(c) depicts the time dependent contact behavior of two adjacent cantilevers. It can be discerned from the figure that the vibration in cantilevers differs in phase of 180° and they make periodic contact at point of maximum deflection. The inset of Fig. 3(c) shows the typical time of contact for two adjacent cantilevers.

It is to be noted that the frequency for operation of this setup is highly dependent on the heat exchange rate^11,33^. A higher heat transfer coefficient will thus enable the system to run at higher frequency. Heat transfer time depends on specific heat, thermal conductivity, volume and mass of cantilevers, which is calculated using the finite element simulation of thermal model^34,35^. Once the heat transfer time is estimated, appropriate frequency of vibration needs to be selected so that there is time for contact between two cantilevers. If this frequency is higher, then insufficient heat transfer is encountered. Similarly, at lower vibration frequency, there is waste of energy to ambient as contact time is greater than required. Hence, optimization of vibration frequency is essential.

**Figure 4:**
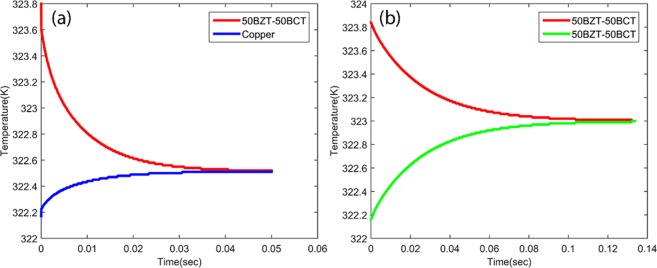
Heat transfer between two thermal bodies over time (**a**) between copper and 50BZT-50BCT, and (**b**) between 50BZT-50BST and 50BZT-50BCT ceramics during cascading.

Hence, it is important to estimate the heat transfer time between two bodies which depend on their density and thermal conductivity. In this study, heat transfer between, (1) sink/source (copper) and elastocaloric patch (50BZT-50BCT), and (2) elastocaloric patches are estimated. Thermal analysis has been done by finite element analysis using material and geometrical properties. Therefore, 2 K temperature difference has been considered between two bodies as an initial condition. Final results for heat transfer as a function of time have been plotted in Fig. 4 (a,b). Figure 4(a) shows the heat transfer time between copper and 50BZT-50BCT is 0.04 s. However, heat transfer time increases to 0.15 s in the case of two 50BZT-50BCT patches are in contact, as shown in Fig. 4 (b). It is obvious due to fact that thermal conductivity of 50BZT-50BCT is 1.8 W/m.K which is lower than that of reported for copper.

**Figure 5:**
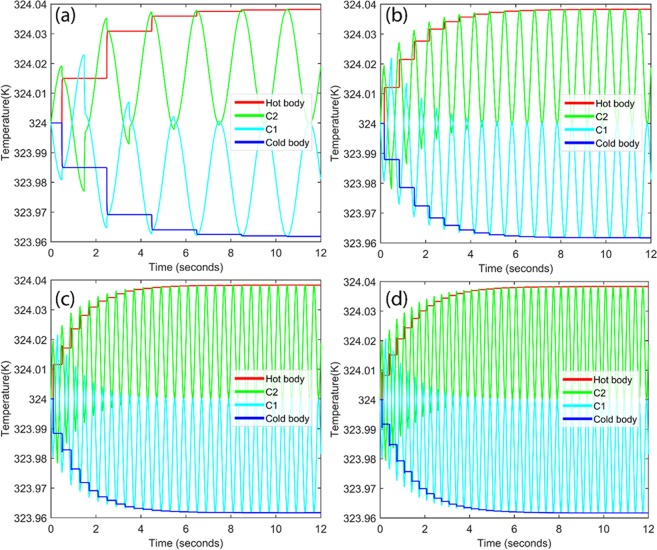
Heat transfer from cold body to hot body for two cantilevers system due to vibration at different frequencies (**a**) 0.5 Hz, (**b**) 1.5 Hz, (**c**) 2.5 Hz, and (**d**) 3 Hz.

**Figure 6:**
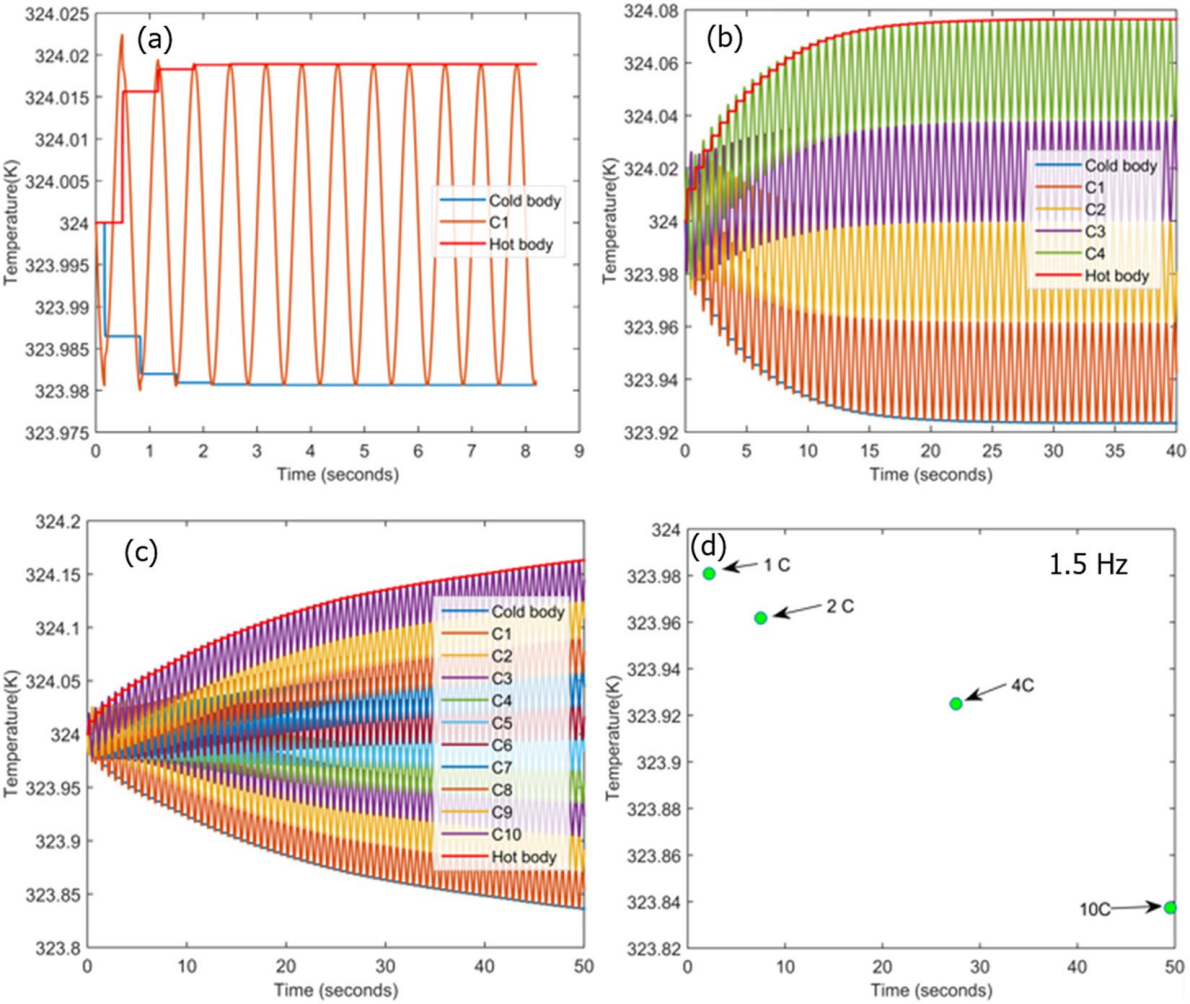
Refrigeration at temperature of 324 K for different number of cantilevers (n) (**a**) n = 1, (**b**) n = 2, (**c**) n = 4 and (**d**) time versus the achieved cooling for different number of cantilevers.

It may be a difficult to hold two cantilevers in contact for 0.15 s to allow complete heat transfer due to their continuous vibrations. However, it can be achieved up to some extent using lowering the frequency of vibrations. It is favorable to select lower vibration frequency because of higher vibration frequency can create impulsive force at the time of contact. For this purpose, first we studied the system for different frequencies 0.5, 1.5 2.5 and 3 Hz, to select optimum frequency. At these frequencies, contact times between cantilevers are 0.042, 0.01, 0.06, and 0.03 s, respectively. It is important to note that possible available contact time is less than the actual heat transfer time (0.15 s). Hence, more number of cycles are required to complete the heat transfer between two cantilevers. Figure 5 shows the heat transfer form cold body to hot body through two cantilevers for different operating frequencies (a) 0.5 Hz, (b) 1.5 Hz (c) 2.5 Hz and (d) 3 Hz. Form Fig. 5, it can be concluded that the higher heat transfer between two bodies occurs at 0.5 Hz however, it takes more time to achieve final thermal equilibrium (~8.6 s). This frequency is not a good option to opt for system as compare to other available frequencies. It can be said that final thermal equilibrium time is about to close each other for 1.5 Hz, 2.5 Hz and 3 Hz. However, it is desirable to select frequency of 1.5 Hz for the system as it generates less impulse force than the other frequencies (2.5 Hz and 3 Hz). Hence, the frequency of operation has been selected as 1.5 Hz. A finite element model was created and its dynamic performance was evaluated with one, two, four and ten cantilevers. Two separate cases were evaluated at 324 K and 358 K as initial operating temperatures, respectively. Figure 6(a) displays the temperature profile for the source and sink, as a function of time with one cantilever in operation. With an initial temperature of 324 K, the first operation cycle produces a net cooling of only ~0.02 K at the source 323.98 K while increasing the sink temperature 324.01 K. Upon the second cycle, the source temperature is further reduced by a small fraction after which thermal equilibrium is achieved and no further cooling is produced. The whole process takes slightly more than two seconds to complete indicating the high response time of the proposed system. Nevertheless, the overall cooling achieved through a single element is negligibly small for practical applications and the system is easily saturated. However, upon increasing the number of cantilevers to two as shown in previous Fig. 5(b), a net cooling of 0.04 K can be achieved which requires slightly over seven seconds. Similarly, when the numbers of cantilevers are increased to four as shown in Fig. 6(b), the source temperature can be effectively reduced from 324 K to 323.92 K over a period of 25 s; while the system appears to be saturated and indicates no possibility of further cooling. Intrigued by these observations, the study was expanded to include 10 cantilevers and the simulation was allowed to run for 50 s. It can be clearly visualized that a steady drop in the source temperature can be observed with a net cooling of about 0.2 K being achieved in slightly more than 50 s, shown in Fig. 6(c). Judging from the slope of the temperature profile, it can be expected that the system will soon attain saturation. Regardless, the results clearly indicate that the proposed system is not only robust and efficient but also highly responsive. Figure 6(d) compares the net refrigeration effect for different number of cantilevers under study. It can be concluded that net refrigeration effect increases with increasing number of cantilevers. However, it also increases the time to reach thermal equilibrium. If there is only then cantilever, temperature of cold body drops to 323.98 K from its initial temperature 324 K in 2.1 s, while for ten cantilevers system, cold body temperature drops to 323.84 K in 50 s. These results indicate an ultra-fast cooling response that can be achieved within a matter of seconds. Additionally, no continual running energy is required as the system can be conveniently brought online almost instantaneously whenever heat dissipation is required and switched off upon completion. Other merits of employing a solid-state refrigerant over conventional halo-carbon fluids have already been discussed^36^.

**Figure 7:**
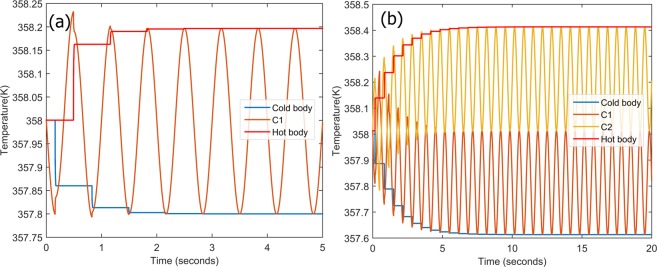
Refrigeration at 358 K for different numbers of cantilevers (n) (**a**) n = 1, and (**b**) n = 2.

Nevertheless, the overall cooling achieved through is system is fairly low (0.48 K) which can be credited to the low adiabatic temperature change capacity of bulk ferroelectrics. One method to address this problem is by designing a system which operates at a crossover of multiple caloric effects[5]. Moreover, external operating parameters also play an important role in determining the performance of bulk ferroelectric materials^34–36^. To demonstrate this phenomenon, we repeated the same set of simulations with an initial operating temperature of 358 K, corresponding to a much larger elastocaloric effect (Fig. 1). Expectedly, the overall cooling capacity of the system was significantly improved as shown in Fig. 7. A single cantilever could cool the system by 0.2 K in approximately one second, while two cantilevers could achieve an effective cooling of 0.4 K. This is essentially the same cooling performance as 10 cantilevers when operated at 324 K, being realized in a fraction of the time. The proposed architecture not only provides a proof of concept but also indicates that fast and effective cooling is possible using bulk ferroelectric materials. The estimated performance is for material parameters of bulk 50BZT-50BCT, and has immense potential for improvement with a higher performance material/configuration combination.

## Conclusions

This study provides possibility of converting mechanical vibration into refrigeration effect. It shows cantilever-based geometry can be employed for heat transfer from cooling body without any separate thermal switch. Vibrations are able to induce cooling through elastocaloric effect associated with ferroelectric materials. For this purpose, bulk *0*.*50Ba(Zr*_*0*.*2*_*Ti*_*0*.*8*_*)O*_3_*−0*.*50(Ba*_*0*.*7*_*Ca*_*0*.3_*)TiO*_*3*_ was selected to simulate the performance using finite element method. Cantilever cascading was also done to increase the net cooling. Further research is warranted to improve the cooling effect as ferroelectric materials are not thoroughly explored for elastocaloric effect.

## Supplementary information

Finite element formulation and its validations
