# Supplementary material for: Vibration induced refrigeration using ferroelectric materials
Source: Sci Rep. 2019 Mar 8;9:3922. doi: 10.1038/s41598-019-40159-8 (PMC6408585; doi:10.1038/s41598-019-40159-8)
Supplement: Supplementary file 1 — Finite element formulation and its validations [file 41598_2019_40159_MOESM1_ESM.pdf]

Vibration induced refrigeration using ferroelectric materials

Anuruddh Kumar[a*], Aditya Chauhan[b], Satyanarayan Patel[c], Nikola Novak[c], Rajeev Kumar[a] and Rahul Vaish[a]

[a]*School of Engineering, Indian Institute of Technology Mandi, Mandi, H.P. India 175 001*

[b]*Department of Materials Science and Metallurgy, University of Cambridge, Cambridge, U.K. CB3 0FS*

[c]*Institute of Materials Science, Technische Universität Darmstadt, 64287 Darmstadt, Germany*

*Corresponding author: anuruddh_kumar@students.iitmandi.ac.in

Appendix A

Using the shape functions, coordinate of any point P, inside the kth layer within the element can be written as

$$\begin{Bmatrix} x \\ y \\ z \end{Bmatrix} = \sum_{i=1}^{nnel} N_i \begin{Bmatrix} x_i \\ y_i \\ z_i \end{Bmatrix} + \sum_{i=1}^{nnel} N_i H_i \begin{Bmatrix} l_{3i} \\ m_{3i} \\ n_{3i} \end{Bmatrix} \qquad \text{(A.1)}$$

where

$$H_i = t_{0k_i} + \frac{\varsigma_k}{2} t_{k_i}$$

Where t_{oki} is the mid plane distance of kth layer from the neutral plane at node i and t_{ki} is the thickness of the kth layer at node. l_{3i}, m_{3i} and n_{3i} are the direction cosines of normal unit vector V_{3i} at node i. If thickness is uniform at each node, then H_i=H. Using the shape functions, displacement of any point, one obtains P inside the kth layer within the element as

$$\begin{Bmatrix} u \\ v \\ w \end{Bmatrix} = \sum_{i=1}^{nnel} N_i \begin{Bmatrix} u_{0i} \\ v_{0i} \\ w_{0i} \end{Bmatrix} + \sum_{i=1}^{nnel} N_i H_i \begin{Bmatrix} l_{1i} & -l_{2i} \\ m_{1i} & -m_{2i} \\ n_{1i} & -n_{2i} \end{Bmatrix} \times \begin{Bmatrix} \alpha_i \\ \beta_i \end{Bmatrix} \qquad \text{(A.2)}$$

where l_{1i}, m_{1i} and n_{1i} are the direction cosine of tangent unit vector V_{1i} and l_{2i}, m_{2i} and n_{2i} are the direction cosine of tangent unit vector V_{2i} at node i. Therefore, each node has 3 translation degree of freedom (u_{oi}, v_{oi}, w_{oi}) in the global coordinates and two rotation degree of freedom (α_i and β_i) about local coordinate. The strain vector ε is defined by the first partial derivative of the displacement vector $[u\ v\ w\ \alpha\ \beta]^T$ by using a differential operator matrix as follows:

$$\{\varepsilon\} = \begin{Bmatrix} \varepsilon_x \\ \varepsilon_y \\ \varepsilon_z \\ \gamma_{xy} \\ \gamma_{yz} \\ \gamma_{zx} \end{Bmatrix} = \begin{Bmatrix} \frac{\partial u}{\partial x} \\ \frac{\partial v}{\partial y} \\ \frac{\partial w}{\partial z} \\ \frac{\partial u}{\partial y} + \frac{\partial v}{\partial x} \\ \frac{\partial v}{\partial z} + \frac{\partial w}{\partial y} \\ \frac{\partial w}{\partial x} + \frac{\partial u}{\partial z} \end{Bmatrix} = [B]\{q\} \qquad (A.3)$$

where $\{q\}_e = \begin{Bmatrix} u_{oi} \\ v_{oi} \\ w_{oi} \\ \beta_i \\ \alpha_i \end{Bmatrix}$ and [B] is strain displacement matrix. Strain energy in element:

$$S.E = \frac{1}{2} \int_{Volume} \varepsilon^T \sigma dV$$

$$S.E = \frac{1}{2} \int_V \left(\{q\}^T [B][C(z)][B]\{q\} \right) dV$$

$$S.E = \frac{1}{2} \left[\{q\}^T \left([K_{uu}] \right) \{q\} \right] \qquad (A.4)$$

where

$$K = \int_V [B][C(z)][B] dV$$

Kinetic energy in element:

$$K.E=\frac{1}{2}\int_V \rho(z)\{\dot{q}\}^{\mathrm{T}}\{\dot{q}\}\,dV$$

$$K.E=\frac{1}{2}\{\dot{q}\}^T[M]\{\dot{q}\} \qquad (A.5)$$

where

$$[M]=\int_V \rho(z)[N]^T[N]dV$$

N are the shape functions. Total external work done on the element:

$$W^s=\int_{S_1}\{q\}^T\{f_s\}\,ds+\{q\}^T\{f_p\} \qquad (A.6)$$

S_1= surface area on which external force is acting, $\{f_s\}$ =surface force intensity, $\{f_p\}$ =point load

$$W^s=\{q\}^T\{F_m\}$$

$$\{F_m\}=\int_S [N]^T\{f_S\}\,dS+[N]^T\{f_p\}$$

By using Hamilton's principle as final equations can be written as

$$\int_{t_0}^{t_f}\delta\left(L+W^s\right)dt=0$$
$$where \quad L=K.E-S.E$$

Using equation A.4, A.5, A.6 and A.7

$$[M]\{\ddot{q}\}+[C]\{\dot{q}\}+[K]\{q\}=\{F\} \qquad (A.7)$$

Appendix B

Dynamic validation of cantilever beam

Geometrical and material parameter of cantilever beam:

Length=100 cm

Width= 10 cm

Thickness= 1 mm

Young's modulus =70 GPa

Poisson ratio =0.3

Density=2700 kg/m^3

Natural frequency of cantilever beam

Analytical solution:

$$f = \frac{\beta_n^2}{2\pi L^2}\sqrt{\frac{EI}{\rho A_c}}$$

Where

Mode Number	β
1	1.875
2	4.694

Present Finite element formulation:

First order shear deformation theory is considered to formulate the thin cantilever beam

Number of element in x direction=50

Number of element in y direction=5

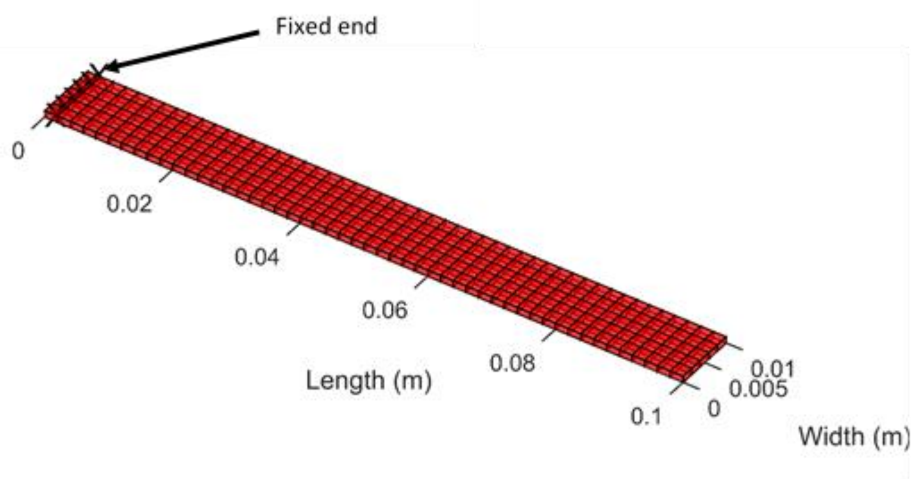

Figure B1: Finite element model of cantilever beam

Comparison of natural frequency (Hz) with analytical solution:

Mode Number	Analytical solution	Present FEM
1	82.24	82.79
2	515.4	519.0

Thermal Analysis Validation:

Present thermal analysis has been validated with exacting reference (Bradeško et al., 2016). All the thermal parameters are considered according the reference and compared the present thermal analysis formulation with the same. Below graph showed the temperature difference after the one cycle for three elements, which show the good agreement with the reference results.

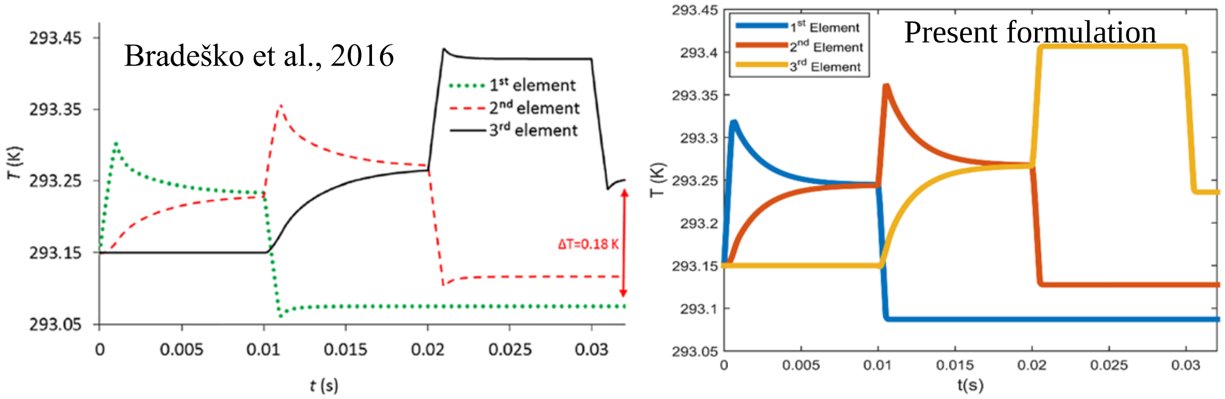

Figure B2: Comparison the results of present formulation with the literature result (Bradeško et al., 2016).

Reference

Bradeško, A. *et al.* Coupling of the electrocaloric and electromechanical effects for solid-state refrigeration. *Appl. Phys. Lett.* **109,** (2016).
